# Triangulating data to define patient pathways, diagnostic and treatment patterns, and outcomes in cancer patients with deep vein thrombosis in Germany: a mixed-methods real-world data study

**DOI:** 10.1007/s00520-025-09840-9

**Published:** 2025-08-14

**Authors:** Vanessa Colonna, Rupert Bauersachs, Roman Gerlach, Roland Jucknewitz, Christoph Kalka, Robert Klamroth, Ulrich Mansmann, Jutta Schimmelpfennig, Mandy Schulz, Martin Tauscher, Helmut Ostermann, Karin Berger

**Affiliations:** 1 Faculty of Medicine, Institute for Medical Information Processing, Biometry, and Epidemiology, Ludwig-Maximilians-University, Munich, Germany; 2 Center for Vascular Research, Frankfurt, Germany; 3 Bavarian Association of Statutory Health Insurance Physicians, Munich, Germany; 4 AOK-Bayern – „Die Gesundheitskasse“, Bereich Exzellenzzentrum Analytik U. Daten, Fachbereich Ökonometrie, Munich, Germany; 5 Vascular Institute Central Switzerland, Aarau, Switzerland; 6 Vivantes Klinikum Im Friedrichshain, Berlin, Germany; 7 AG Thrombose DGPL, Aktionsbündnis Thrombose, Freiburg, Germany; 8 Central Research Institute for Ambulatory Health Care in Germany, Berlin, Germany; 9 Ludwig-Maximilians-University Hospital, Munich, Germany; 10 University of Cologne, Cologne, Germany

**Keywords:** Cancer incidence, Mixed-methods analysis, Thrombosis

## Abstract

**Purpose:**

Cancer incidence is rising in Germany, increasing the burden of cancer-associated deep vein thrombosis (DVT). To improve prevention, diagnosis, and treatment, robust regional data on patient numbers, cancer types, healthcare access, and outcomes are essential but currently scarce. This study addresses these gaps using a multi-source approach.

**Methods:**

A mixed-methods analysis was conducted using claims data (2016–2018) from the Bavarian Association of Statutory Health Insurance Physicians and AOK Bayern. Inclusion criteria: age > 18 years, active cancer diagnosis (ICD-10-GM C00–C99), and incident thrombosis (ICD-10-GM I80–I82). A supplementary patient survey captured care access and travel times.

**Results:**

Among 677,327 Bavarian cancer patients, 38,393 (6%) developed thrombosis (mean age 69.6; 56% female). DVT occurred most frequently in skin cancers (30%), breast (16%), and digestive organs (16%). Complications were documented in 8610 patients, including pulmonary embolism (9%) and chronic venous disease (23%). Hospitalization occurred in 34% of cases (men, 29%; women, 39%). Mortality averaged 9% for cancer patients and rose to 23% when thrombosis was present. Rural patients reported average travel times of 53 min (range, 15–250) to specialized centers.

**Conclusion:**

Cancer-associated thrombosis presents a significant clinical burden, especially in common tumor types. High rates of complications and mortality, combined with limited access to specialized care—particularly in rural areas—underline the urgent need for targeted prevention, better care coordination, and education strategies based on real-world evidence.

## Background

In Germany, a large number of people suffer from venous thrombosis each year or are at risk of developing deep vein thrombosis (DVT) and/or pulmonary embolism [1] due to hereditary factors (mutations and polymorphisms of coagulation factors) or acquired factors (e.g. cancer, use of contraceptives). The number of deaths associated with DVT and pulmonary embolism (PE) in Germany is estimated to exceed 40,000 per year [2]. Thrombosis is one of the most common and dangerous complications for cancer patients [3–5]. The overall risk of DVT in patients with cancer is approximately up to nine times higher as in the general population, depending on the type, stage, and treatment of the tumour [6–8]. Patients with cancer-associated thrombosis have a higher risk of death, major bleeding, and recurrence than patients without cancer do [9]. With the number of incident and prevalent cancer cases projected to rise significantly in Germany in the near future, a corresponding increase in cases of cancer-associated deep vein thrombosis (DVT) is expected. This highlights the urgent need to focus attention—both from the patient and healthcare provider perspectives. In this context, supportive care like prevention and treatment of thrombotic events, particularly with regard to innovations in cancer treatment, often remains underemphasized. Given the multitude of complex decisions involved in the treatment of cancer, the prevention and management of disease- and therapy-related complications, such as DVT, are not the initial focus of doctor-patient discussions. However, knowledge about the risk of thrombosis, its early signs, and, most importantly, the necessity of compliance and adherence to prescribed prophylactic or therapeutic treatment is of critical importance. Noble et al. showed that the adherence of patients with cancer and thrombosis and their awareness of the condition were dependent on the quality of education provided by the treating physician. The underlying study showed that cancer patients received insufficient information about their cancer-associated risk for DVT and that awareness of the importance of treatment was low [10]. In today’s context improvement of health literacy to empower individuals in managing their health effectively and enhancing treatment compliance and adherence is a high-priority topic for patient organizations, healthcare professionals, health insurers, and policymakers nationally and internationally.

Another factor that impairs outcomes in the care of cancer patients with thrombosis in Germany is the fragmented responsibility for treatment and the frequent lack of interdisciplinary, up-to-date, and rapidly accessible information exchange among healthcare professionals. A survey of physicians (DGHO, BNHO, and DGO) showed that more than half of the haematologists/oncologists delegated the management of thromboembolic events to general practitioners and angiologists/phlebologists [11]. Optimizing information exchange between healthcare professionals is particularly important in federally organized healthcare systems such as Germany’s, where outpatient and inpatient care, as well as general practitioners and specialists, are structurally separated. Fragmented responsibilities across medical disciplines and regionally diverse healthcare infrastructures further complicate care coordination—an essential factor for the effective management of patients with cancer and thrombotic complications. To address these challenges, transsectoral and interdisciplinary communication—potentially supported by digital tools—is crucial and is currently being explored through various initiatives.

A fundamental prerequisite for improving healthcare structures is the development of clear patient pathways and the precise definition of responsibilities among healthcare providers. To assess patient pathways, the current state of healthcare provision, including the patient’s perspective on healthcare services in different regions, is key. On this basis, new concepts of care can be developed to sustainably secure and improve healthcare especially in fragmented healthcare systems.

Therefore, this study examined routine cancer care in different regions of Bavaria in terms of patient journeys, diagnostic and treatment patterns, clinical outcomes based on claims data, and patient perspectives.

## Methods

### Study design

This study used a mixed-methods design. Quantitative and qualitative data were collected and analyzed. The study included a secondary data analysis of the Statutory Health Insurance database of the Bavarian Association of Statutory Health Insurance Physicians, KVB, the claims database of the AOK-Bayern Die Gesundheitskasse, one of the biggest statutory health insurance in Bavaria, and an online patient survey.

### Secondary data analysis

#### Statutory health insurance data

SHI data were used to determine the demographic and clinical characteristics, diagnosis and treatment modalities, and clinical outcomes in terms of subsequent events (pulmonary embolism, post-thrombotic events, and bleeding). The database comprises outpatient contractual medical billing data from the KVB and drug prescription data. Data from the 2016–2018 period were analyzed. The inclusion criteria for cancer patients were age ≥ 18 years with at least one malignant disease according to the International Classification of Diseases, German Modification (ICD-10-GM) C00-C97, documented in at least two quarters per year (M2Q). The inclusion criteria for patients with cancer and DVT were documented DVT diagnosis (ICD-10-GM I80, I81, I82) between 2016 and 2018 (index quarter), no documented DVT for at least two quarters prior to the DVT index quarter, and malignant disease (see above) within 1 year (four quarters) prior to or after the DVT index quarter.

#### AOK Bayern—“Die Gesundheitskasse” data

The AOK Bayern data were analyzed using the SHI data. Due to the availability of inpatient data, additional information on deaths and hospitalisations was collected. The mortality rate was defined as the number of documented deaths in 2016–2018. The hospitalisation rate was defined as hospitalisation in 2016–2018, with DVT (I80 I82) as the documented main diagnosis and reason for hospitalisation.

#### Regions

The settlement structure district types of the Federal Institute for Research on Building, Urban Affairs, and Spatial Development were used to divide Bavaria into regions [12]. For characterising large and medium-sized cities, the proportion of the population is used, as well as the population density of the district region and the population density of the district region excluding large and medium-sized cities. By this, Bavaria is divided into four types of regions: large cities (at least 100,000 inhabitants), municipal districts (large and medium-sized cities of at least 50% and a population density of at least 150 inhabitants/km^2^, as well as districts with a population density without large and medium-sized cities of at least 150 inhabitants/km^2^), rural districts with densification approaches (districts with a population share in large and medium-sized towns of at least 50%, but a population density of less than 150 inhabitants/km^2^, as well as districts with a population share in large and medium-sized towns of less than 50% and a population density excluding large and medium-sized towns of at least 100 inhabitants/km^2^), and sparsely populated rural districts (population share in large and medium-sized towns of less than 50% and a population density excluding large and medium-sized towns of less than 100 inhabitants/km^2^).

### Patient survey

A web-based, anonymous 37-item survey was conducted. The survey was reviewed by medical experts from the BEQUEST project, representatives of the psycho-oncology department of Medical Department III of Ludwig Maximilians University Hospital, and the Bavarian Cancer Society. The final questionnaire has been pilot tested by three patients. The survey was conducted over 11 months. SPSS version 26 (IBM, Somers, NY, USA) was used for the descriptive data analysis.

#### Regions

The categorisation of the region in the results of the patient survey is based on the inhabitants. The categorisation deviates from the regions observed in the secondary data due to the utilisation of patient responses in the categorisation process. It was only feasible to inquire about the proportion of inhabitants residing in the place of residence, as the collection of more abstract data, such as density, is not well-suited for an online survey.

## Results

### Secondary data analysis

Table 1 indicates that the proportion of insured individuals with cancer across all regions is approximately 6%. In rural districts, both densely and sparsely populated, there are about 53,000 more SHI-insured individuals with documented cancer diagnoses than in large cities or municipal districts. Around 18% more women than men have a history of both cancer and thrombosis diagnoses. The most common tumours in patients with cancer and DVT included melanoma and other malignant neoplasms of the skin (35), malignant neoplasms of digestive organs (16%), and breast cancer (16%). Approximately one in 18 cancer patients will develop DVT. Patients with both cancer and DVT were, on average, 1 year older than those without DVT.

**Table 1:** Demographics and clinical characteristics of the SHI patient population based on KVB outpatient data and subgroup analysis: AOK-Bayern–insured patients; based on AOK-Bayern data, in- and outpatient billing data

	Large city	Municipal district	Rural district with densification approaches	Sparsely populated rural district	Total
Cancer patients Bayern (SHI 2016–2018)					
Number of cancer patients, total	137,056	162,311	187,754	190,206	677,327
Male	59,874 (44)	75,986 (47)	89,856 (48)	90,363 (48)	316,079 (47)
Female	77,182 (56)	86,325 (53)	97,898 (52)	99,843 (52)	361,248 (53)
Age range Mean Median	19–105 68.65 72	19–108 68.81 71	19–106 68.53 71	19–108 68.68 71	68.7
Patients with cancer + DVT (SHI 2016–2018)					
Patients with cancer + dvt, total	8261	8984	10,816	10,332	38,393
Male	3223 (39)	3725 (41)	4481 (41)	4228 (41)	15,657 (41)
Female	5038 (61)	5259 (59)	6335 (59)	6104 (59)	22,736 (59)
Mean age (median)	70.65 (73)	69.96 (73)	69.21 (71)	69.37 (72)	69.6
Subsequent events in patients with cancer + DVT (within 15 months of dvt diagnosis)					
Pulmonary embolism (i26), (% of cancer + dvt)	735 (9)	805 (9)	954 (9)	920 (9)	3448 (9)
Other venous diseases (i87) (incl. post-thrombotic syndrome)	1848 (22)	2011 (22)	2436 (23)	2315 (22)	8610 (23)
Bleedings (i60–i62)	81 (1)	90 (1)	106 (1)	103 (1)	383 (1)
SUBgroup: cancer patients Bayern (AOK-Bayern 2016–2018)					
Number of cancer patients, total	43,778	55,305	82,998	91,466	273,547
Male	20,518 (47%)	26,611 (48%)	40,165(48%)	44,014 (48%)	131,308 (48%)
Female	23,260 (53%)	28,694 (52%)	42,833 (52%)	47,452 (52%)	142,239 (52%)
*Mean age (*± *SD)*	68.44 (± *14.31)*	69.90 (± *13.78)*	69.66 (± *13.60)*	69.38 (± *13.68)*	69.41
Death (cancer) (2016–2018)					
*Total*	3900 (8.9%)	4568 (8.3%)	3.848 (9.6%)	7920 (8.7%)	23,502 (8.6%)
Male (26)	2,172,172 (10.6%)	2518 (9.5%)	3.266 (7.6%)	4293 (9.8%)	12,831 (9.7%)
Female (26)	1728 (7.4%)	2050 (7.1%)	3.266 (7.6%)	3627 (7.6%)	10,671 (7.5%)
Patients with Cancer + DVT (AOK-Bayern 2016–2018)					
Patients with cancer + DVT, total	2455	2852	4436	4663	14,406
Male (26)	1118(46%)	1257(44%)	1932 (44%)	2083 (45%)	6390 (44%)
Female (26)	1337 (54%)	1595 (56%)	2504 (56%)	2580 (55%)	8016 (56%)
*Mean age (*± *SD)*	70.32 (± 13.38)	71.63 (± *12.79*)	71.22 (± *12.73*)	71.22 (± *12.58*)	71.15
Hospitalization with DVT*					
Total (26)	897 (37%)	943 (33%)	1494 (34%)	1541 (33%)	4875 (34%)
Male (26)	481 (43%)	489 (39%)	748 (39%)	780 (37%)	2498 (39%)
Female (26)	416 (31%)	454 (28%)	746 (30%)	761 (29%)	2377 (29%)
Death (patients with cancer + DVT)*					
*Total*	586 (24%)	637 (22%)	994 (22%)	1110 (24%)	3327 (23%)
Male (26)	321 (29%)	322 (26%)	469 (24%)	575 (28%)	1687 (26%)
Female (26)	265 (20%)	315 (20%)	525 (21%)	535 (21%)	1640 (20%)

^*^In the index quarter, up to three quarters after the index quarter

Table 1 also indicates that within the AOK-Bayern dataset across all regions, male patients with both cancer and DVT are hospitalized more frequently than female patients. From 2016 to 2018, 34% of patients with cancer and DVT required hospitalization. Patients in the AOK-Bayern dataset are, on average, 1 year older than those in the KVB dataset.

Table 2 shows that D-dimer levels are infrequently measured in outpatient settings, with general practitioners being the primary users of D-dimer testing. Diagnostic imaging for DVT is performed most frequently in large urban areas. Oncologists and hematologists conduct only a limited number of DVT diagnostic procedures and are therefore excluded from this analysis.

**Table 2:** Physician groups performing outpatient image diagnosis or prescribing medication for patients with cancer + DVT patients stratified by physician residency. *n* describes the number of patients treated

Large city	*n*	Municipal district	*n*	Rural district with densification approaches	*n*	Sparsely populated rural district	*n*
Imaging diagnostics							
Vascular surgery	1,693	Vascular surgery	313	Internal medicine	639	Internal medicine	459
Surgery	950	General practitioner	286	Vascular surgery	315	Surgery	339
General practitioner	282	Internal medicine	241	General practitioner	289	Cardiology	277
Medication							
General practitioner	3886	General practitioner	4444	General practitioner	5204	General practitioner	5,225
Vascular surgery	665	Internal medicine	164	Cardiology	320	Internal medicine	292
Angiology	478	Vascular surgery	136	Internal medicine	248	Surgery	190
D-dimer							
General practitioner	383	General practitioner	354	General practitioner	555	General practitioner	492
Trauma Surgery	< 30	Internal medicine	< 30	Internal medicine	35	Internal medicine	37
Internal medicine	< 30	Haematology/oncology	< 30	Angiology	< 30	Haematology/oncology	< 30

### Patient survey

**Fig. 1:**
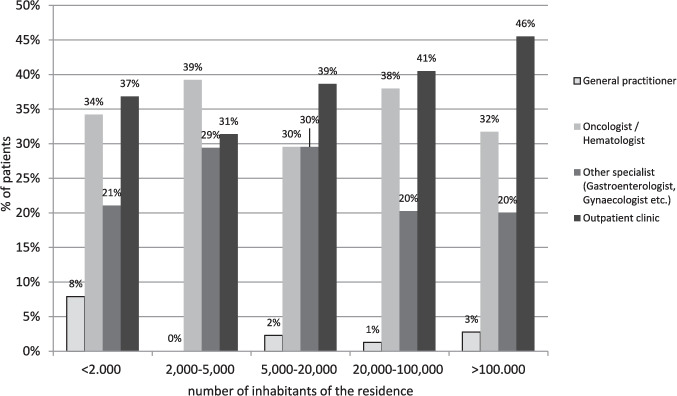
Answers to the patient survey to the question: Who performs/performed the cancer therapy? Who is your main contact person? Stratified by the number of inhabitants of the place of residence; *N* total = 409

Figure 1 illustrates that specialist outpatient clinics and specialists administer cancer treatment and serve as primary points of contact for cancer patients. Over 40% of individuals from more densely populated regions (> 20,000 inhabitants) utilize specialized outpatient clinics as their main medical contact. A small proportion of patients identified general practitioners as their main contacts, with the highest percentage being 8% in regions with fewer than 2000 inhabitants. Haematologists/oncologists were named as the main contact by approximately 30–39% of patients and other organ specialists by 20–30%.

Table 3 demonstrates that the distance to clinics was, on average, the longest for hospitals and the shortest for general practitioners. The primary caregivers for cancer treatment are specialist outpatient clinics and specialists, who serve as the main points of contact for patients. Those residing in large cities had shorter distances from their primary points of contact.

**Table 3:** Mean journey to physician time in minutes

	> 100,000 Inhabitants	20,000–100,000 Inhabitants	5000–20,000 Inhabitants	2000–5000 Inhabitants	< 2000 Inhabitants	Total
General practitioner	15.15 (*n* = 148)	13.49 (*n* = 82)	12.19 (*n* = 89)	12.76 (*n* = 50)	13.6 (*n* = 38)	13.7 (*n* = 412)
Haematologist/oncologist	27.37 (*n* = 136)	30.9 (*n* = 73)	35.4 (*n* = 81)	35.00 (*n* = 49)	32 (*n* = 39)	41.7 (*n* = 382)
Hospital	32.53 (*n* = 149)	43.3 (*n* = 79)	52.3 (*n* = 86)	41.7 (*n* = 50)	53 (*n* = 42)	42 (*n* = 410)

## Discussion

By triangulating multiple data sources, the BEQUEST study is one of the first German investigations that provides evidence on the number of cancer patients with deep vein thrombosis (DVT) in routine care, focusing on Bavaria, where approximately 16% of the German population resides and 87% of the population is statutory health-insured. Between 2016 and 2018, in a population of about 677,327 statutory insured persons with a cancer ICD coding, a total of 38,393 incident DVT cases with a mean age of 69,6 years were documented. In other words, approximately one in every 18 cancer patients is diagnosed with a new DVT. Thrombotic events and the associated diagnostics, treatments, complications, and outcomes in terms of mortality are currently only sparsely recorded in regional cancer registries or in the German national cancer registry. This study may serve as an example for national and international researchers, demonstrating the potential of administrative claims data analyses to continuously address the aforementioned information gaps using up-to-date data from routine oncological care.

The most common entities where DVT has been identified in this population-based study were melanoma and other malignant neoplasms of the skin, malignant neoplasms of the mammary gland, and malignant neoplasms of the digestive organs. This is slightly different when compared to epidemiology study results. While tumors with a high thrombotic risk, such as pancreatic and brain cancers, show elevated relative incidences of DVT, the majority of cases in absolute numbers occur in more common cancers like breast, prostate, and colon due to their higher prevalence. The observation that DVT incidence was 20% higher among women with cancer must be interpreted in the context outlined above and may, in part, be attributable to the fact that, during the study period, approximately 12% more female than male cancer patients were living in Bavaria.

Findings from the BEQUEST study underscore the importance of integrating real-world data to identify patient groups in routine care with the highest burden of cancer-associated DVT. Such insights are essential to guide the development and implementation of targeted preventive and educational strategies within everyday oncology practice, particularly to address complications related to DVT.

Further, this study demonstrates that DVT-associated complications and outcomes in terms of mortality are significant in populations of patients with cancer. At least for one in five patients with cancer and DVT complications such as pulmonary embolism (9%) and post-thrombotic events (23%) were documented. These results align with findings in the literature, where 4–20% of cancer patients experience pulmonary embolism at some point [12]. Our findings regarding pulmonary embolism are notable, albeit slightly lower than those reported in the literature. In cancer patients with deep vein thrombosis (DVT), the incidence of subsequent pulmonary embolism (PE) varies depending on the study population, cancer type, disease stage, treatment modalities, and individual risk profiles. However, published data generally estimate that approximately 10–30% of cancer patients with DVT develop a PE within the first weeks to months following the initial thrombotic event [13]. Data from the RIETE registry and other literature demonstrated that fatal PE was the second most common cause of death and ahead of common cancer-related morbidities (respiratory insufficiency and infection) [14]. Our findings on post-thrombotic syndrome are also in line with published literature [15, 16].

It is plausible to assume that complications attributable to DVT contribute to a substantial clinical burden in cancer patients, leading to both physical and psychological impairment and further compromising their health-related quality of life. The mortality of cancer patients with DVT is significantly higher compared to patients without venous thromboembolism (VTE) 8.6% vs. 23%. Both the underlying malignancy and the thromboembolic complication (DVT, potentially accompanied by pulmonary embolism) contribute to this increased risk. The mortality rate in our study was 9% higher compared to the general population.

Examining patient pathways and diagnostic treatment patterns is key to understanding real-world care and identifying gaps in service provision. In Germany, the healthcare system is organized at the federal rather than national level. Patients with cancer and DVT can be managed by various healthcare professionals, and patients are free to choose which physician or special centre or hospital to consult. The availability of specialists in Bavaria varies significantly between regions [17]. Patients in urban areas generally have better access to haematologists and oncologists than those in rural regions. For example, in 2022, Upper Franconia East—a predominantly rural area—had just under 92,000 inhabitants per haematologist/oncologist, whereas Munich, a large urban center, had approximately 46,500 inhabitants per specialist [17]. The patient survey showed that patients prefer to be treated by office-based specialists and specialised centres in clinics and are willing to travel up to 250 min to do so.

The specialization of office-based physicians involved in the care of patients with DVT has been found to be highly heterogeneous, but antithrombotic medications were mainly prescribed by general practitioners in all regions. Actually, in the German healthcare system, there is no structured process on sharing clinically relevant information between healthcare professionals on a patient level. Improving structural processes to enable timely and low-threshold communication is therefore currently a central topic of ongoing discussions, particularly regarding the use of digital solutions for the exchange of treatment-related information. The hospitalization rate with DVT as the primary reason for admission was 39%.

Data generated in the BEQUEST study provide valuable insights into the healthcare professionals involved in the care of patients with cancer and DVT across different regions and help identify potential target groups for educational interventions or discussions aimed at developing improved care structures.

Moreover, the analyses revealed regional differences in diagnostic and treatment pathways between urban and rural settings. The BEQUEST data revealed that imaging procedures are predominantly performed in large cities, potentially limiting diagnostic access for elderly or less mobile individuals living in rural or underserved areas.

D-dimer diagnostics, which are part of a guideline-based diagnostic regimen, were rarely documented in the used database. This represents an important contribution of this claims data analysis, as it highlights—based on real-world data—the need to raise awareness of D-dimer testing, particularly in the office-based setting. Furthermore, it should prompt discussions on which measures could facilitate the successful implementation of current guidelines into routine clinical practice.

## Limitations

The secondary analysis of claims data on the care of patients with cancer and deep vein thrombosis (DVT) in Bavaria is subject to several general limitations when using administrative claims data. Most notably, the accuracy of recorded diagnoses and services data depends on coding quality and their relevance for reimbursement purposes. Additionally, the absence of detailed clinical information—such as tumor stage in terms of TNM and Eastern Cooperative Oncology Group (ECOG) performance status or number of line of therapies—limited the assessment of clinical characteristics. This information would be essential for more granular in-depth analysis, e.g. regarding complications, mortality, and interpretation. This study restricted its analysis to deep vein thrombosis (DVT) as the inclusion criterion, given the availability of a specific ICD code for DVT, in contrast to the broader category of cancer-associated thrombosis (CAT), for which no distinct coding exists. Consequently, it is likely that the actual burden of thrombosis-related complications among cancer patients in routine care is substantially underestimated in this dataset.

Due to the quarterly structure of outpatient administrative claims data, it was not possible to determine the precise temporal sequence between the DVT diagnosis and the initial cancer diagnosis. Nevertheless, the robustness of the data was enhanced by incorporating both pre- and post-observation periods. Despite these limitations, claims data offer valuable real-world insights into large patient populations. The patterns and signals identified through this approach are crucial for guiding further research and identifying areas in need of targeted interventions. Whether the differences in the specialties involved lead to different outcomes or whether and how these groups of doctors work together on an interdisciplinary basis cannot be deduced from secondary data and requires further study. While claims data lack detailed clinical variables, they provide important signals.

## Conclusion

The substantial number of cancer patients with deep vein thrombosis (DVT) identified in Bavarian claims data—along with the frequent occurrence of complications such as pulmonary embolism and post-thrombotic syndrome, as well as elevated hospitalization and mortality rates—highlights the considerable burden of DVT in this population. This study further highlights the importance of complementing epidemiological risk assessments with data on the actual prevalence of DVT among patients with cancer. Notably, in the BEQUEST study, the most frequently documented malignancies were skin neoplasms, malignant neoplasms of the breast, and digestive organ cancers—rather than pancreatic cancer and other malignancies typically associated with higher thrombotic risk in epidemiological research. These BEQUEST findings underscore the urgent need to increase awareness of DVT numbers and the DVT-associated burden to ensure the implementation of appropriate preventive measures and diagnostic procedures and strengthen patient health literacy. Anticipated demographic changes, the rising incidence and prevalence of cancer, regional epidemiological disparities, and the centralization of medical expertise in urban areas pose significant challenges to the equitable delivery of high-quality, needs-based healthcare. At the same time, digital innovations may offer promising opportunities to address these issues and support more effective, decentralized care solutions.

Given that cancer registries often lack detailed information on supportive care–related complications, routine analyses of healthcare claims can provide valuable insights into the real-world burden and management of cancer-associated DVT. Therefore, ongoing monitoring using claims data is a valid approach to generate insights and develop models for needs-based healthcare. Moving forward, linking claims data with clinical and registry data will be essential to support more comprehensive analyses and inform targeted interventions for patients with cancer and DVT. This is especially relevant in the context of novel oncologic therapies, where real-world evidence on toxicity and side-effect management remains scarce.

Unlike clinical studies, analyses of healthcare delivery in specific regions require a strong national focus, as healthcare systems and reimbursement structures differ and can significantly influence access to diagnostics and treatment. Nevertheless, it is essential that national findings are shared internationally. Cross-border knowledge exchange is crucial to foster mutual learning and to support the development of care models that ensure equitable access to optimal treatment for patients with cancer and DVT.

## Data Availability

Data are not publicly available. Data was analyszied by employees of the AOK Bayern or ZI.
